# Mentorship Strategies and Illustrative Cases at Every Career Stage

**DOI:** 10.1002/aet2.70131

**Published:** 2026-02-16

**Authors:** Adrianne N. Haggins, Ryan Walsh, Arthur T. Broadstock, Katrina A. Gipson, Marianne T. Haughey, Pamela Dyne, Maria Moreira, Robert M. Rodriguez, Elizabeth E. Leenellett, Richelle J. Cooper

**Affiliations:** ^1^ Department of Emergency Medicine University of Michigan Ann Arbor Michigan USA; ^2^ Department of Emergency Medicine Vanderbilt University Medical Center Nashville Tennessee USA; ^3^ Department of Emergency Medicine University of Cincinnati Cincinnati Ohio USA; ^4^ Zucker School of Medicine at Department of Emergency Medicine Long Island Jewish Medical Center/Northwell New York USA; ^5^ Medical Center Department of Emergency Medicine Olive View‐University of California Los Angeles (UCLA) California Los Angeles USA; ^6^ Department of Emergency Medicine, Denver Health and Hospital University of Colorado School of Medicine Denver Colorado USA; ^7^ Department of Medicine, School of Medicine University of California at Riverside Riverside California USA; ^8^ Department of Emergency Medicine, David Geffen School of Medicine at UCLA University of California at Los Angeles (UCLA) California Los Angeles USA

## Abstract

**Background:**

Introspection and guidance shape an individual's academic path. Mentorship plays an essential role in intrapersonal development as well as organizational growth.

**Methods:**

We present a literature synthesis and case scenarios based on a didactic sponsored by the Academy for Diversity and Inclusion in Emergency Medicine (ADIEM), the Association of Academic Chairs of Emergency Medicine (AACEM), and the SAEM Faculty Development Committee delivered at the Society for Academic Emergency Medicine (SAEM) 2025 Annual Meeting.

**Results:**

Mentors' guidance, coaching, and sponsorship are essential at all career stages. Successful mentorship is grounded in the mentee's goals. Mentee self‐reflection and communication are key to defining career goals and strategies for success at all career stages, whether in education, research, administrative, or operations. Mentorship can help early‐career faculty translate areas of interest into scholarship and advancement and can help mid‐career faculty with transitions to leadership positions, as well as mitigate career burnout and enhance satisfaction. Among late‐career faculty, mentorship can facilitate a shift toward legacy building, opportunities for growth, and new skill development. Mentors are key allies to help faculty pivot at any stage to ensure goals align with personal satisfaction.

**Conclusions:**

Impactful mentorship requires communication, self‐discovery, and adaptability. Mutual respect and active engagement with a trusted mentor build the bridge to achieve a career vision and advance the specialty.

## 1 Introduction

Mentorship relationships are integral for professional development given their positive effect on an individual's career satisfaction, scholarly productivity, and career advancement [1]. Mentees report these relationships enhance their clinical and leadership skill development, as well as their ability to secure research funding [2]. Mentorship has moved from reliance on a single “guru” to networks [3] of supportive and influential persons with complementary roles (e.g., near‐peers, late career mentors, career advisors, coaches, and sponsors) [4]. Organizations benefit from mentorship by growing the department and building leadership capacity, which contributes to the teaching mission and professional identity formation of learners [2]. These key relationships represent a commitment to foster a community that invests in the success of the next generation and furthers the practice of medicine.

Women [5, 6, 7, 8, 9] and those from backgrounds underrepresented in medicine (URIM) often face barriers to advancement related to a paucity of mentors, experiences of discrimination, isolation, “minority tax,” [10, 11, 12] and disproportionate caregiving responsibilities [13]. Other factors such as first‐generation in college [14], socioeconomic status (SES), and generation [15, 16] can present challenges navigating academic culture. Shared research or clinical interests, as well as the intersection of identities and lived experiences, influence mentorship connections. Non‐URIM mentors may choose a colorblind approach in their mentoring [17]. However, women [5] and racial minorities [18, 19] mentees may prefer identity concordant mentors who understand how marginalized identity(‐ies) affects professional experiences. Within generation cohorts (see Table 1) [20, 21, 22, 23, 24, 25, 26, 27, 28, 29], there may be relatable childhood experiences, work traits, teaching and learning styles, as well as expectations for interactions and communication [15, 16]. Best practice is to openly discuss expectations as these generalizations can lead to implicit biases or assumptions. Awareness of a mentee's or mentor's background can deepen the relationship, and it can also become a barrier if someone feels unprepared to engage effectively across different identities.

**TABLE 1:** Intergenerational cohorts.

	Lived experiences	Academic experiences
Baby Boomers (1945–1964)	Civil rights, TV, Vietnam war, economic prosperity; “Traditional” family unit	Educational hierarchy, lecture‐based education
Gen X (1964–1980)	Economic pessimism, computers; Non‐traditional families	Independent learners, problem‐solvers
Millennials (1980–1997)	Terrorism, 9/11, multiculturalism, technology boom; Helicopter parents	Team‐based educational environment, Internet as source material
Gen Z (1997–2012)	Post‐9/11, economic downturn, COVID pandemic, social media, political activism Smaller families, incr. Dx prevalence of mental health and learning differences	Digital natives, videos > textbooks, value creativity and less hierarchy in learning/teaching

Academic careers characteristically advance through stages (e.g., early, mid‐career, late) which present some shared and unique challenges to navigate. These stages are defined based on time since initial faculty appointment: early (0–8 years), mid‐ (> 8 years) [30], and late career phase approximates to more than 15–20 years. To cultivate productive mentorship relationships, it is important to consider the common personal and professional hurdles that present at these different career stages. We describe mentoring approaches to address challenges during early, mid, and late career stages. We present illustrative cases to highlight strategies that empower mentees and mentors, while recognizing these issues can arise at any point in a career.

## 2 Methodology

A collaborative group of 10 faculty from across the country delivered a mentorship workshop “Faculty Development Throughout the Career Spectrum,” at the Society for Academic Emergency Medicine (SAEM) 2025 Annual Meeting. The session was sponsored by the Academy for Diversity and Inclusion in Emergency Medicine (ADIEM), the Association of Academic Chairs of Emergency Medicine (AACEM), and the SAEM Faculty Development Committee. The faculty represented a diverse coalition including Assistant, Associate, and Full Professors with areas of academic focus spanning education, operations, and research, as well as individuals who identify as under‐represented in medicine. The faculty included vice chairs of research and faculty affairs, former program directors, a former designated institutional official (DIO), chairs of faculty development, as well as certified professional coaches. The interactive session combined brief didactics and case‐based scenarios to engage small group discussions focused on: mentoring best practices, special populations, intergenerational dynamics, and transitions across career stages. The faculty developed the content through an iterative process, first by brainstorming ideas based on other forums and professional experiences, then gathering literature on best practices for coaching and mentorship. Faculty were assigned to the didactic sections to create a presentation and break out session cases using the career stage framing. Roles included a section lead for each career stage and 2–3 other faculty assigned as small group facilitators. All the faculty provided edits and revisions to the content and cases. Approximately 80–100 individuals across the career spectrum, from trainees to department chairs, attended the didactic session and participated in case discussions. Immediately following the session, the faculty debriefed key observations and insights. Approximately 1 month later, the group reconvened to synthesize personal stories and lessons learned from the small group discussions. This manuscript integrates the literature review with themes from the break‐out and large group discussions, highlighting cases and concepts that resonated most with the attendees.

## 3 Identifying Mentee's Goals and Clarifying Expectations

Mentorship should maintain consistent focus on the mentee's goals. In this paper, the term mentorship is used broadly to include mentors and others who provide guidance such as advisors, sponsors, and coaches defined in Table 2 [31, 32, 33, 34, 35]. Each career stage will be defined by the mentee's vision and their decisions reflecting: What now? What next? And with whom? A deliberate approach guides the longitudinal mentee‐mentor relationship by encouraging the mentee to articulate their interests, values and priorities. This provides the basis for developing the mentee's personal mission statement and creates a foundation for mentee‐defined metrics of success, as well as which professional development opportunities and networks to target [36]. Honest self‐assessment of one's current skill set and the skills one desires to develop (e.g., be a better bedside teacher, a better lecturer, effectively run a committee) is key to professional growth.

**TABLE 2:** Defining and distinguishing guidance roles.

	Purpose	Directionality of relationship	Situations when best to go to…	Giving advice is…	Applied example: When faced with a challenge
Coaching	Encourage self‐determination, reflective thinking, self‐discovery, and self‐efficacy. Gain new insights, clarity, and confidence	Egalitarian	Personal and/or professional development	Avoided	A coach would ask, “Which of your strengths will help you in overcoming this challenge?”
Advising	Provide advice based on advisor's area of expertise	Hierarchical	Professional guidance	Warranted	An advisor might say, “This is my wheelhouse, X is your best way forward here”
Mentoring	Provide mentorship based on mentor's experience	Hierarchical	Professional guidance	Expected	A mentor might say, “Based on what I've seen over the years and know about you, I think you should consider doing X”
Sponsor	Support career advancement through offering resources and position of influence	Hierarchical or egalitarian	Professional guidance	Expected	A sponsor may say, “There is an opportunity to lead this committee, and I can mention your name if you are interested?”
Teaching	Instruct a learner involving a specific curriculum	Hierarchical	Improve knowledge and/or skills	Welcomed	A teacher might offer, “Here's a method that has been studied of how to approach it”
Therapist	Manage a mental health condition	Hierarchical	Manage a mental health condition	Sought after	A therapist might say,” “What feels most threatening to you in your current situation and what would feel most supportive to you right now?”

Open and honest communication is a key aspect to achieving mentee and mentor goals at all stages. A structured framework—including clear objectives, roles, and expectations—can be instrumental in creating successful relationships. Some formal mentoring programs utilize agreements or mentorship contracts [37]. The structure imposed and length of relationship will vary based on the individuals and tasks (e.g., career guidance versus a grant submission). An academic research career has specific objectives and timelines which benefit from a structured focus and mentorship plan to increase success. These special considerations can be found in the Figure S1. Across career paths and stages, there are personal real‐life challenges that affect career trajectory and work‐life integration, some of which are planned while others are unexpected such as caring for family (childcare, eldercare, etc.), family planning, illness or disability. While our cases focus on themes at various stages, we recognize career pace varies and issues can arise at any stage.

## 4 Special Considerations for Women and Individuals Underrepresented in Medicine

Academic women EM physicians report lower rates (36.2% vs. 47.5%) of formal mentorship than men [38]. Participant satisfaction with career advancement and access to opportunities is highest among those with formal mentorship compared to those with informal or no mentorship [38]. Facilitating connections through institutional or national professional development groups can enhance access to support networks of peers and mentors, as well as increase visibility [39]. Strategies to address limited mentorship for women physicians include structured group mentoring programs [5], and peer mentoring groups led by a trained facilitator [40] or a senior mentor [41, 42]. Online forums, such as social media, websites, or blogs can increase access to peer communities for career development [42] and space to amplify voices of persons underrepresented [43] in academic medicine. Faculty advocates that create transparency about successful promotion processes and related institutional faculty development are important allies for women and URIM faculty [44].

URIM faculty face similar and unique challenges identifying mentors as women EM physicians. Building relationships with faculty outside of the department/institution (e.g., distance mentoring model) is an option for both groups [44, 45]. Effective mentorship does not mean the mentor and mentee have to share identities. In a systematic review of URIM mentoring programs, discordant mentor pairs were not associated with reduced mentee satisfaction [46]. Mentor preparation through workshops and materials was a key component to these programs [46]. Fostering cross‐identity relationships is not without its hurdles [47, 48]. Supporting the careers of those URIM requires understanding the “chilly climate” that can contribute to negative experiences for racial minorities [49]. Mentorship that draws attention to race, mistrust, and understanding Black men's experiences in and outside academic medicine fosters deeper, more successful mentoring [50]. Mentors should develop an openness to inquire about mentees' preferences as mentees may choose to “strategically suppress” less visible identities (i.e., first‐generation, SES, etc.) [51].

## 5 Mentoring Across the Career Stages

### 5.1 Early Career

The early career is often recognized as the most amorphous stage; a time with flexibility for career exploration. The transition from resident learner to faculty supervisor is an exciting and challenging time as individuals seek to define their professional identity. Failing to define one's career goals can lead early career faculty to be pulled in multiple directions. Some individuals may have a very defined focus and not yet have the mentors and sponsors they need. Getting started, particularly when new to an institution, includes identifying mentors, resources, and collaborators. Peer and late career faculty mentoring relationships that develop organically from shared interests or are assigned by the department or institution can be crucial to success.

Academic promotion requires an understanding of institutional and departmental values (e.g., teaching, service, grants, scholarship, mentorship, awards) [52]. Matching these requirements to one's academic interests leads to personal satisfaction and success. Pololi's academic development plan (ADP) [53] and the SAEM Academic Promotion Toolkit [54] can guide career choices. Balancing being a “good citizen” in the department (e.g., serving on committees, teaching courses or lectures to students and residents), growing an academic portfolio, and maintaining healthy work‐life integration requires learning to appropriately accept and decline potential opportunities. A mentor, coach, and sponsor can help the mentee navigate this balancing act. Illustrative case examples and mentoring strategies for supporting early career faculty are provided in Table 3.

**TABLE 3:** Early career mentorship example cases.

Background	Challenge	Stakes	Strategy			Why it matters
			Mentee	Mentor	Institution	
**“The Ultimate Juggle”—**Dr. A is a single parent in Year 3, splitting their time between clinical, teaching residents, and serving on two hospital committees	**Feels overwhelmed**—late nights grading simulation scenarios, too many committee calls, and never enough family time. Teaching, scholarship, and networking feel impossible	Risk of burnout, strained family relationships, and potential impact on promotion	**Evaluate goals with Mentor:** Either independently or with a personal development coach, develop a values‐based personal vision statement. Use it to decide which work aligns with that vision and stop doing work that does not advance it **Align** committee, teaching, mentorship and scholarship‐ make it count twice or more (a lecture locally, then regionally or nationally in a webinar, and write it up)	**Time‐Block Coaching:** Helps carve out “scholarship sprints” (e.g., 2 h Tuesday mornings) and negotiates fewer committee assignments **Accountability Partner:** A peer mentor in a similar life stage for monthly “balance check” to re‐adjust commitments	**Support:** explore institutional or departmental needs that link to the mentee's mission or vision that may provide some protected time **Provide** opportunities for personal development coaching **Discuss** with the Chair whether decreasing FTE would be feasible	Sustainable work‐life integration bolsters long‐term career success and personal well‐being
**Finding Your Niche & Network”—**First‐generation immigrant and the only physician of color on faculty, Dr. B is in Year 1 and interested in global health	**Feels isolated**—few collaborators locally, no formal path to build their interest into a defined role. Unsure who “looks like them” in leadership	Without a supportive network, Dr. B worries they'll drift into “general service” without academic focus	**Define global EM goals:** Seek out external mentors via national organizations to help determine their true interests, be it building infrastructure, building EM education capacity, global research, and if there is a specific region of focus? **Define a “Micro‐Niche” Project:** Mentor helps them launch a pilot tele‐EM program with a sister hospital abroad, giving them ownership and visibility **Funding:** identify funding sources to ensure viability of the project long term	**Connect to External Mentors:** Introduce to affinity groups (i.e., Global Emergency Medicine networks) for role models, community, and collaboration Seek faculty connections in the region that Dr. B wants to work, and with other national organizations or EM clinicians working in the area (e.g., SAEM Global Emergency Medicine Academy (GEMA)) For any global work, bidirectional mentorship and establishing partnerships in the other country are key	**Regular Sponsorship Check‐Ins:** Quarterly meetings with the Department Chair or another faculty advocate to discuss priorities, champion their advancement, and address barriers to progress	Focused mentorship accelerates both personal fulfillment, accountability, and likelihood of retention
**“Shaping a Research Niche from Diverse Interests”—**In their second year as an EM faculty, Dr. C has published on everything from sepsis biomarkers to ED crowding to medical education. They are excited by new ideas and have an inbox full of “would you like to join this study?” invitations	**Breadth without depth**—Their curriculum vita (CV) reads like a buffet; they haven't carved out a “go‐to” area that grant reviewers can see them as a developing expert. They have worked with many but have no stable mentorship team. Their research output, though broad, lacks depth in any one domain. As a middle author on multiple projects, they have not established themselves as a first author	Without a clear, fundable niche, Dr. C risks stalled grant applications, diluted academic identity, and potential difficulty with academic promotion timelines	**Phased Pipeline Plan:** Outline a 12‐month plan; Phase 1 (Q1‐Q2): Focus exclusively on a small pilot grant around agreed on niche Phase 2 (Q3‐Q4): Submit IRB protocol and begin data gathering	**Priority Matrix Workshop:** Mentor sits down with mentee to map their interests on a two‐axis chart—“passion vs. funding potential”—and identifies one or two high‐value areas **Accountability Check‐Ins:** Monthly “pipeline huddles” to ensure mentee isn't drifting into side studies and to celebrate small wins **Planning:** Help mentee create a timeline and strategy for grant submission, first author publications and didactics in their selected niche	**Support Focused Faculty Development:** Offer protected time through internal grant opportunities, or seed funding tied to project goals to help faculty like Dr. C make the leap from generalist to specialist **Streamline Research Support Services—**Help provide centralized grant writing help, IRB navigation support, and biostatistics consults	By finding a niche it enables the mentee to increase fundability, impact, identity, and sustainability

Regardless of academic track (e.g., education, research, administration, etc.), the early career faculty member should identify one or two clinical (or operational) content areas to focus their interest. This is important to efficiently navigate academic promotions and/or obtain grant funding. The ultimate goal is to develop a national or international reputation as an expert. An area of focus creates synergy between one's passion and academic obligations to facilitate scholarship and advancement. For example, serving on the hospital quality improvement committee for pediatric sepsis can lead to lectures on pediatric sepsis and publications. A network of mentors including individuals within one's own institution and externally (e.g., regionally, nationally, internationally) allows access to a range of perspectives to inform continued self‐reflection, growth, and success (as defined by the individual faculty member) throughout one's career. The late career mentor(s) should facilitate professional connections and sponsor appointments or positions based on the early career mentee's goals.

### 5.2 Mid‐Career

The mid‐career stage capitalizes on the developing areas of expertise and can result in new professional or leadership opportunities. During this phase, there is often an expectation of academic promotion from Assistant to Associate Professor. This requires a comprehensive evaluation of one's teaching, administrative, service, and scholarly productivity. This process can prompt a desire to pursue new directions or reveal a need for growth. At mid‐career, many find challenges arising from balancing their professional and personal responsibilities. Professional discontent can result from unmet or unclear ambition. Table 4 highlights cases illustrating mid‐career challenges and mentorship approaches.

**TABLE 4:** Mid‐career mentorship example cases.

Background	Challenge	Stakes	Strategy			Why it matters
			Mentee	Mentor	Institution	
**“Career 2.0”**—In the process of preparing for academic advancement, Dr. D, realizes that while they have been teaching, writing, and serving on various committees, they are not passionate about their work. They have an idea for a large research project, but don't know how to get started and are embarrassed to ask their research colleagues for help at this stage in their academic career	**Building Focus and Confidence**—Dr. D needs to develop new research skills and establish a clear focus, overcome self‐doubt by seeking help, identify new mentors and collaborators, and reallocate current responsibilities to create time for these efforts	Without a mentor to assist in defining their new focus and help them skill‐build, their success is not assured. Without changing focus, they risk burnout and career stagnation due to lack of engagement in their non‐clinical academic activities	**Identify Research Mentorship:** Talk with research faculty (locally or through collaboration with other national groups) about their interests and identify a mentor (or mentorship team) **Align Responsibilities with Goals:** Speak with chair about changing career focus and request removal from committees that are not in keeping with their interests. Request protected time to pursue new goals while explaining how developing expertise, pilot data, or future grant submissions will advance departmental academic output, reputation, or funding **Manage Burnout & Uncertainty**—Personal development coaching strategies for managing their decision‐making process, concerns about burnout and career uncertainty	**Support:** The mentor meets the faculty member where they are, tailoring support to their interest and experience as they pursue a new research focus **Start Small:** Help mentee realize they need to gain research skills with a smaller project first. This can result in obtaining pilot data, gaining additional research mentors and a team that can then feed into developing the larger project they want to pursue	**Support** faculty development opportunities (e.g., master's in public health, American College of Emergency Physicians (ACEP) Advanced Research course or SAEM Advanced Research Methodology Evaluation and Design (ARMED) course) **Provide** scalable options based on available institutional resources, ranging from more structured research support services (centralized grant writing help, IRB navigation support, and biostatistics consults) to informal cross‐departmental mentoring and ad hoc statistical consultation **Provide** opportunities for personal development coaching **Ensure the Research** Director has the resources needed to assist interested faculty, regardless of their academic rank.	Mentee may face burnout and loss of purpose from unfulfilling work, leading to a desire to shift academic focus. Often lacking skills for this transition, they benefit from targeted mentorship and personal coaching to support growth and sustain a meaningful career
**“The balancing act**”—Dr. E, a mid‐career Assistant Program Director (APD), must decide whether to accept a long‐desired Program Director (PD) role while also navigating and supporting their child's recent medical diagnosis and related academic and social challenges	**Navigating Work‐Life Demands—**With significant competing priorities, Dr. E must consider whether they can realistically balance their family needs with the demanding PD role	If they do not accept the offer to become PD at this time, will there be other opportunities to advance their career in the future? If they accept the PD job, will they be able to be present for their family as they navigate uncertain times and unknown future challenges? Either choice may ultimately lead to regret	**Craft Personal Vision**: Either alone or with a personal development coach, create a vision statement for themselves, which incorporates their values at this time in their life **Find Trusted Advisor:** Identify a trusted and knowledgeable advisor who could help brainstorm what life as the PD could look like in their department **Align Goals:** Discuss with the co‐parent/support system their goals and expectations for care of their newly diagnosed neuro‐diverse child	The trusted advisor helps brainstorm ways in which they could do both, that is, what would be needed from the rest of the leadership team and department for Dr. E to be successful as PD and also have the flexibility needed to be present with their family on their terms **Provide** the space for mentee to explore what else they might want to do if they choose NOT to take the PD job in favor of having more time available for their family **Help** mentee explore if they have “all‐or‐none” thinking and to challenge this mindset	**Provide** space and time for Dr. E to figure out what they want. Work creatively with Dr. E to support them assuming the PD role, for example, could an additional APD share the workload? Could additional administrative support protect more time for Dr. E and the team? **Provide** a safe and supportive environment to decide, recognizing the challenge of the decision itself	Dr. E's career progression as well as their meaningful participation in their family are at risk. Personal circumstances can change, destabilizing a previously anticipated career goal Academic departments can and should work creatively and collaboratively with their faculty to allow personal and professional goals to co‐exist and evolve while also meeting departmental needs
**“The Ceiling Effect.”**—Dr. F, a mid‐career associate medical director (AMD), would like to move up to become an ED director, however no such position is available at their institution	**Exploring New Opportunities**—Dr. F has a specific career goal that is not available in their current department. Career advancement will require reframing their goals to a different leadership/operations position within their institution or seeking a new job at a different hospital	Dr. F risks career stagnation, boredom and burnout if they do not make a big shift, either in career goals or career location	**Create Personal Vision:** Either alone or with a personal development coach, create a vision statement for themselves, which incorporates their own personal values at this time in their life. Focus on whether their vision of themselves is tied more to location or anchored more to their role **If role driven**: Seek guidance on finding a new position aligned with their goals **If location driven:** Seek guidance on deciding if remaining as AMD is in keeping with their goals or if there is another role that would be more fulfilling	**Help** Dr. F clarify whether their long‐term goals are flexible or tied specifically to the ED director role **Explore** potential leadership opportunities outside their current institution that align with their vision **Support** them in reframing success to include growth beyond title—through mentorship, new projects, or system‐level roles. Provide sponsorship and connections if seeking a position at a different hospital	If Dr. F decides they want to remain in their current department but in an expanded or entirely different role, then providing opportunities to discover what they find engaging is essential, even if that means reimagining existing roles and responsibilities	Individuals may feel like they are trapped in a role that they have outgrown and hold themselves back from exploring options due to lack of clear personal vision. Academic departments can and should allow space for individuals' personal growth within their department, or risk losing them to another institution

The mid‐career phase presents unique opportunities for faculty to seek leadership roles and growth that leverages the skills they have acquired. These opportunities and/or desire for further skill development may involve relocation. Mid‐career is also a stage at which gender disparities in the workforce are amplified. Women emergency physician attrition occurs at an increased rate and earlier in their career than men. Attrition, as well as other gender disparities, contribute to lower rates of women emergency physicians in leadership roles and in the rank of professor [55]. In 2020, women represented 50% of the healthcare workforce, but were only 22% of professors, 18% of department chairs, and 17% of medical school deans [56]. Sponsorship, mentorship, and institutional initiatives are critical to address the gender disparities that negatively impact women emergency medicine physicians.

Coaching strategies [57] such as appreciative inquiry [58, 59], unconditional acceptance, facilitating a growth mindset [60, 61], and goal setting for accountability can help mitigate feelings of discontent. As faculty members consider changing their academic or administrative focus, it may be helpful to revisit their personal mission statement or to create a vision statement with the help of a coach, trusted mentor, or peer. This self‐reflective process can clarify goals and identify internal or external resources, learning opportunities, and leadership interests/roles. A comprehensive guide such as the Clinician Educator Milestones [62] can serve as a roadmap for new skill development. Additional validated self‐assessment tools include VIA Institute on Character [63] and Strengthfinders [64], as well as in popular press books focused on core values, such as “Dare to Lead” by Brene Brown [65].

### 5.3 Late Career

During the late career, faculty have established their reputation and track record of achievement in their area(s) of expertise. At this stage, faculty are coveted for leadership roles and shift their career focus toward legacy‐building. Mentoring others can provide the late career faculty member a renewed sense of purpose, as well as the opportunity to share wisdom, both personal and institutional, accumulated over the course of their career [66]. Mentorship opportunities through professional organizations can lead to new connections and contributions to the department/organizations, thereby decreasing burnout [67]. Late career faculty are uniquely positioned to advocate for meaningful change in academic medicine, precisely because of the social capital gained through their established reputation, access to leadership, as well as reduced concern about career advancement. This makes them powerful allies [68, 69] to advance novel and inclusive organizational recruitment and retention practices. Their voices and faculty development committees [70] can amplify calls for systemic reform to model the values of justice and belonging for the next generation.

This later phase may also pose challenges in *finding* mentors. As personal and professional priorities change, it is imperative to engage in self‐discovery for new or reimagined roles. Faculty may struggle with maintaining continued career satisfaction, keeping current with medicine and technology, managing health concerns, planning transitions for retirement or succession planning, clinical scheduling, and work‐life integration related to care for elders or adult children with special needs [71, 72, 73, 74, 75] (see Table 5 for case examples). Mentorship from a peer or groups can provide support and insightful approaches for these transitions [4]. National organizations in emergency medicine and external to emergency medicine (e.g., Association for American Medical Colleges (AAMC), etc.), as well as institutional and national faculty development programs can serve as resources for finding guidance and mentors. Reverse mentorship [76] is another opportunity that fosters meaningful relationships by allowing late career faculty to learn from early career colleagues with expertise in emerging medical practices or technologies. This approach helps late‐career faculty stay current, adapt to new advancements, and facilitate their continued growth mindset.

**TABLE 5:** Late career mentorship example cases.

Background	Challenge	Stakes	Strategy			Why it matters
			Mentee	Mentor	Institution	
**“Elder care”**—Dr. G, a late career faculty member, is navigating the stress of having their critically ill mother recently admitted to their own hospital's ICU. After her discharge, Dr. G becomes overwhelmed by a wave of calls to coordinate home care, social work, and visiting nurse services while balancing work responsibilities	**Dual Responsibilities:** Balancing family and professional responsibilities. Communication challenges and stress of dealing with colleagues and other specialties from the family member perspective	Dr. G, risks being perceived as “unprofessional” as they try to balance care of their family as well as work responsibilities. These extra responsibilities may raise concerns from colleagues about their mental and physical health	**Set clear boundaries:** Communicate with your work and family's healthcare teams about specific windows of unavailability to manage family logistics without compromising patient care **Leverage available resources:** Request social work, case management, or employee wellness support to assist with care coordination **Prioritize self‐care:** Acknowledge emotional toll and seek time for rest, reflection, or counseling to prevent burnout	**Encourage** Dr. G to take the necessary time off or adjust their responsibilities to enable them to care for their family **Normalize the challenge:** Reassure Dr. G that this is a common and understandable struggle, not a sign of unprofessionalism. Recommend resources such as family leave or employee assistance programs **Provide emotional support:** Offer space to process the dual identity of provider and caregiver and validate the stress of navigating both roles	Ensure there is a clear, **stigma‐free process** for emergency family leave or short‐term duty adjustments that is inclusive of paid time off to care for elders, not just children **Streamlined** pathways to access elder care options (care coordination support, wellness services, and peer support) through the institution	Caring for family includes elder care, which relies on a fragile patchwork system often unnoticed until physicians face it firsthand. Without support, this burden can lead to burnout, perceived unprofessionalism, and loss of late career faculty engagement
**“Health issues”**—Physical limitations are impairing Dr. H's ability to mobilize around the department. Dr. H needs a surgery but is concerned that if they take the appropriate time off they will be found less relevant to the department	**Supporting Late Career Faculty:** Developing priorities within the department to allow for aging in the professional venue. Having the department value the skill set brought by late career faculty	Loss of late career faculty leads to both departmental gaps and diminished income and professional identity for the faculty member	**Legal & HR Protections:** Evaluate the protections afforded through HR and federal legislation **Time:** Allow the late career faculty time to heal **Financial Planning Support:** Develop financial planning skills to decrease the anxieties of taking time from work	**Mentor Role:** This is a situation in which the mentor doesn't need to be a physician. The mentor can be someone from HR or familiar with HR policies **Expertise:** The mentor should understand the rules and financial aspects of taking extended sick or disability leave **Emotional Support:** The mentor should also help the mentee cope with the loss of personal value as they step back from clinical duties	Provision of clearly stated and discoverable HR policies to support sharing of knowledge in this area	Health challenges can affect anyone at any time, although more likely to occur in late career Clear policies and supportive measures around illness and sick leave can reduce anxiety and help extend faculty careers
**“What next?”‐** Dr. J, isn't feeling joy in their current role. They find they sit in too many meetings, are overwhelmed with night shifts and feel undervalued in their current role Additionally, Dr. J feels uncomfortable with using point‐of‐care ultrasound, digital media, and evolving educational models leading to reduced connection with learners further contributing to dissatisfaction	**Balancing Security and Fulfillment:** The lack of updated skills, meaningful connection with learners, and clarity about career options can be daunting	Loss of skilled faculty, job dissatisfaction, depression, decreased productivity and disengagement	**Self‐Assessment:** Mentee needs to self‐assess needs and wants (finances) and other options for career paths **Evaluate Skills:** Explore whether new skills may need to be acquired. Pursue targeted training in point‐of‐care ultrasound, digital media, and updated teaching models **Advocate:** Request fewer night shifts and reduced low‐value meetings	**Facilitate** finding multiple peers in disparate fields to provide perspectives and discuss alternate career choices Help Dr. J **identify core values** and what aspects of work still bring energy or meaning. Help Dr. J explore options to adjust responsibilities **Connect** Dr. J with faculty development resources and define set goals regarding new skill acquisition	**Providing career path opportunities** for late career faculty as they adjust clinical duties based on physiologic needs or interest **Recognize and reward non‐clinical contributions** such as leadership, mentorship, teaching, or innovation **Provide robust faculty developmen**t resources to facilitate skill development and maintenance throughout the career spectrum. A supportive environment allows for all stage faculty to be engaged as a lifelong learner	When late career faculty like Dr. J lose joy, burnout and turnover can follow. Supporting self‐assessment, skill growth, and exploring new career paths helps retain talent and boost fulfillment

## 6 Deciding When to Pivot

At any point along the career trajectory, one may consider whether to pivot by switching academic tracks, administrative roles, or areas of focus. Understanding how to manage career transitions is a growing area of interest [77, 78]. “Pivoting” [79] is defined as keeping one foot steady and shifting the other foot into a new area. Career pivoting realigns one's goals with changing life priorities to increase flexibility in work schedule, reconsider professional commitments, or explore a new passion. This process starts with intentional self‐reflection by taking inventory of current skills and roles. This allows faculty to decide what inspires them and brings satisfaction [77, 80]. The next step is defining a new role that leverages those identified skills or presents an opportunity to expand those skills. This also provides clarity into which tasks or roles should be shed.

A trusted mentor or coach can help explore strengths, opportunities for development, and pivoting. For example, a program director might consider becoming a Dean of Undergraduate Medical Education or a Designated Institutional Official (DIO) [3]. Those seeking lifestyle changes may seek additional certification (e.g., obesity medicine, addiction medicine, professional coaching, etc.), insurance company work, or other non‐clinical sectors that do not require night or weekend work [81]. Transitions have potential beneficial or deleterious implications on compensation and responsibilities, as well as unforeseen consequences. Mentors can help identify some of these issues and strategize negotiation. This often requires finding mentors outside of the mentee's usual circle, possibly non‐academic, depending on career next steps. National meetings or organizations can expand access to mentors and networks with relevant expertise. The American Association for Medical Colleges (AAMC) may be a resource for those pursuing an administrative or educational dean's role in the medical school. The Accreditation Council for Graduate Medical Education (ACGME) can be a resource for those looking to become a Designated Institutional Officials (DIO). Those interested in hospital administrative leadership positions may consider additional degrees such as a master's in business administration or healthcare administration, which can facilitate connections to new mentorship networks.

## 7 Mentoring Partnerships That Inspire Exploration, Growth, and Empowerment

Transformative mentorship centers on relationship‐building, character development, and inspiration [82], rather than merely transferring skills or knowledge. As emphasized in our SAEM 2025 session, the mentorship relationship should be grounded in authentic connection, mutual respect, and chemistry that nurture exploration and innovation. Being a good mentee is about showing up with your vision and strategically engaging with a network of people invested in your growth and achievements. Mentors and the aims of mentoring will shift over time with evolving personal and professional focus. Mentorship should be mutually beneficial to the mentor and mentee. Mentees should “manage up” to promote clear communication with mentors and demonstrate ownership of their career objectives [83]. This is fostered when mentors listen, resisting the urge to overstep, and recognize the mentee's agency and value in defining their career path [84]. Maintaining engagement involves understanding that mentorship should not mimic hierarchical management and requires adapting to evolving needs, communication styles, and schedules [85]. When barriers arise—such as misaligned expectations, communication gaps, or lack of availability—address them early through open, respectful dialogue.

Communication requires being able to listen to the other person's perspective, striving to achieve mutual understanding, even when you do not agree. For both parties to grow, it is essential to demonstrate a willingness to learn different ways of doing things and resist the urge to become upset. It is important to extend grace, because there will be instances where a mentor or mentee does not work in the way expected, and that should not necessarily end the relationship. Mentorship creates an opportunity to share your approach of how you do things and why it works for you, not to replicate yourself or impose your ideals on your mentee. Effective teams, whether in the emergency department or in mentoring dyads/groups, work to everyone's strengths. Mentorship is about empowering the mentee to obtain knowledge and skills to achieve success and develop into the best version of themselves.

Career trajectories are not always linear. Mentees should be encouraged to reassess and pivot their goals when appropriate. Transitions are not only acceptable but often necessary for adaptability, long‐term fulfillment, and growth. A robust and complementary mentor network—composed of individuals who offer varying perspectives and expertise—can be instrumental in navigating the career transitions, as they open doors to new opportunities, guidance, and support. Ultimately, providing high‐quality mentorship not only advances individual careers and their professional contentment but also strengthens the broader academic ecosystem by retaining talent and promoting a culture of collaboration.

## Author Contributions

All authors contributed to the concept and design of the manuscript, drafting of the manuscript, and critical revision of the manuscript for important intellectual content. For this manuscript, acquistion of data, analysis and interpretation of data, statistical expertise and acquistion of funding were not applicable.

## Funding

The authors have nothing to report.

## Disclosure

The conceptual synthesis of mentorship, faculty development and interactive cases described in this paper was presented at the 2025 SAEM Annual Meeting in Philadelphia.

## Conflicts of Interest

A.N.H. receives grant funding from NIH (NHLBI and NINDS) for unrelated clinical research and advising on a data safety monitoring board. R.M.R. receives grants from NIAID, CDC, NINDS, and Pfizer. R.J.C. receives grant funding from NIH (NHLBI and NINDS) and PCORI for unrelated clinical research. R.J.C. receives a stipend from the American College of Emergency Physicians (ACEP) for editorial services provided at *Annals of Emergency Medicine*. R.W., A.T.B., K.A.G., M.T.H., P.D., M.M. and E.L. have declare no conflicts of interest.

## Supporting information


**Figure S1:** Research pathway mentorship.

## Data Availability

Data sharing not applicable to this article as no datasets were generated or analysed during the current study.
