# Supplementary material for: Mentorship Strategies and Illustrative Cases at Every Career Stage
Source: AEM Educ Train. 2026 Feb 16;10(1):e70131. doi: 10.1002/aet2.70131 (PMC12908424; doi:10.1002/aet2.70131)
Supplement: Supplementary file 1 — Figure S1: Research pathway mentorship. [file AET2-10-e70131-s001.pdf]

Figure 1. Research Pathway Mentorship

Planning and mentorship are instrumental for successful careers in research. The research path requires a skill set that includes generating scholarly work, obtaining grant funding and managing budgets/teams, skills that are not often obtained prior to completion of residency. The timeclock for early career award mechanisms, e.g., K awards can be catalysts to productive research careers. Structured and consistent mentorship is essential with input from faculty with expertise as clinician-scientists who have been independently funded. These mentors do not necessarily need to be in emergency medicine.

Early Career

Mentor

Delineate research goals and interests early
Consider group mentoring via research advisory teams
Assess knowledge and skill limitations and have lists of *go-to* people for certain topics
Help identify funding sources
Guide negotiating protected time
Provide honest feedback and support regarding feasibility
Emphasis on the fact there is no single correct path

Mentee

Create detailed research career plan and systematically write an **Early Career Research Agenda Methods Paper**

Introduction-Describe research interests and the why?

Objectives-Outline specific research career goals

Methods- Detail specific activities and milestone timeline

Limitations-Anticipate challenges and contingency plans

Mid Career

Mentor

Continue candid review of successes, challenges, and lessons about how to navigate transitions
Consider sponsoring mentee to be part of grant review committees or NIH study sections
Maintain emphasis on delineation of goals, group mentoring, provide referrals and give honest feedback

Mentee

Draft a **Mid Career Research Agenda Methods Paper** (same as early career) and a **Research Agenda Results Paper**

Results-What did they accomplish towards their original goals

Discussion/Limitations-What worked, what didn't work, and why?

Conclusions-Discussion about their path forward – next research agenda

Late Career

Mentor

Guidance is often focused on self-direction, peer-to-peer mentoring, and commonly shifting attention to advising and supporting the next generation of clinician scientists.

Mentee

Lessons Learned Paper/ Lecture:

Summarize methods from previous papers-What did they do, and how?

Review of results-Findings from papers, grants, and other achievements

Discussion-What advice would they give to early career faculty?

Future personal and communal agenda-Plans for leadership transfer in ongoing research programs
